# Early differentiation of mesenchymal stem cells is reflected in their dielectrophoretic behavior

**DOI:** 10.1038/s41598-024-54350-z

**Published:** 2024-02-21

**Authors:** Ioan Tivig, Leslie Vallet, Mihaela G. Moisescu, Romain Fernandes, Franck M. Andre, Lluis M. Mir, Tudor Savopol

**Affiliations:** 1 Biophysics and Cellular Biotechnology Department, Faculty of Medicine, Carol Davila University of Medicine and Pharmacy, 8 Eroii Sanitari Blvd., 050474 Bucharest, Romania; 2 Excellence Center for Research in Biophysics and Cellular Biotechnology, Carol Davila University of Medicine and Pharmacy, 050474 Bucharest, Romania; 3 METSY UMR 9018, Université Paris-Saclay, CNRS and Gustave Roussy, 94805 Villejuif, France

**Keywords:** Mesenchymal stem cells, Dielectrophoresis, Differentiation, Cell separation, Adipogenic, Osteogenic, Lab-on-a-chip, Stem-cell research, Mesenchymal stem cells, Biological physics

## Abstract

The therapeutic use of mesenchymal stem cells (MSCs) becomes more and more important due to their potential for cell replacement procedures as well as due to their immunomodulatory properties. However, protocols for MSCs differentiation can be lengthy and may result in incomplete or asynchronous differentiation. To ensure homogeneous populations for therapeutic purposes, it is crucial to develop protocols for separation of the different cell types after differentiation. In this article we show that, when MSCs start to differentiate towards adipogenic or osteogenic progenies, their dielectrophoretic behavior changes. The values of cell electric parameters which can be obtained by dielectrophoretic measurements (membrane permittivity, conductivity, and cytoplasm conductivity) change before the morphological features of differentiation become microscopically visible. We further demonstrate, by simulation, that these electric modifications make possible to separate cells in their early stages of differentiation by using the dielectrophoretic separation technique. A label free method which allows obtaining cultures of homogenously differentiated cells is thus offered.

## Introduction

Since their discovery by Fridenstein and col.^1^, the mesenchymal stem cells (MSCs) were shown to be characterized by a high capacity to self-renew and a great ability to be differentiated not only into osteocytes, chondrocytes, and adipocytes (which represents a criterium for being denominated MSCs)^2,3^ but also in cells belonging to ectodermal (neuron-like) and endodermal (hepatocytes, β pancreatic cells) lineages^4,5^. Nowadays, due to their differentiation capabilities, which are of interest for cellular replacement procedures, MSCs are used in a wide range of applications, mainly in tissue engineering and in regenerative medicine^6–12^. The interest for MSCs therapeutic use increased also because of their immunomodulatory capabilities^13–15^. It was shown that, in vivo, the undifferentiated MSCs protect other delicate cells which are co-transplanted with them^16^.

Specific protocols are applied to get different cell types starting from MSCs. Often these protocols are lengthy and MSCs differentiation is incomplete, leading to mixtures of cells in different stages of differentiation (including undifferentiated MSCs)^17,18^. Therefore, development of efficient methods to purify the MSCs progenies of interest is mandatory.

Dielectrophoresis (DEP) is a non-destructive method which is used in cell sorting techniques^19^ and analysis of certain electrical parameters^20^. In practice, the DEP force occurs when applying a non-uniform AC electric field that manipulates the motion of a particle (e.g., a cell), based on the difference between the polarizabilities of the particle and the suspension medium. A dielectric particle suspended in an inhomogenous (AC) electric field experiences a force given by^21^:

$$\vec{F}_{DEP} = 2\pi r^{3} \varepsilon_{0} \varepsilon_{med} Re\left[ {K\left( f \right)} \right]\vec{\nabla }\left| {E^{2} } \right|$$

where $r$ is the particle radius, $\varepsilon_{0}$ and $\varepsilon_{med}$ are the electric permittivities of the vacuum and suspending medium, respectively, and $\vec{\nabla }\left| {E^{2} } \right|$ is the gradient of the electric field intensity; $Re\left[ {K\left( f \right)} \right]$ is the real part of the Clausius–Mossotti factor and reflects the polarizability properties of both the particle and medium:

$$K\left( f \right) = \frac{{\varepsilon_{p}^{*} - \varepsilon_{med}^{*} }}{{\varepsilon_{p}^{*} + 2\varepsilon_{med}^{*} }}$$

where ${\varepsilon }_{p}^{*}$ and ${\varepsilon }_{med}^{*}$ are the complex electric permittivities of the particle and suspending medium, respectively. In the case of a particle composed by a homogenous core surrounded by a shell (membrane), which is a good approximation for a biological cell, it has the following expression:

$$\varepsilon_{p}^{*} = \varepsilon_{mem}^{*} \frac{{\left( {\frac{r}{r - d}} \right)^{3} + 2\left( {\frac{{\varepsilon_{int}^{*} - \varepsilon_{mem}^{*} }}{{\varepsilon_{int}^{*} + 2\varepsilon_{mem}^{*} }}} \right)}}{{\left( {\frac{r}{r - d}} \right)^{3} - \left( {\frac{{\varepsilon_{int}^{*} - \varepsilon_{mem}^{*} }}{{\varepsilon_{int}^{*} + 2\varepsilon_{mem}^{*} }}} \right)}}$$

where $d$ is the membrane thickness and $\varepsilon_{int}^{*}$ and $\varepsilon_{mem}^{*}$ are the complex electric permittivities of particle interior and shell (membrane).

As mentioned above, $Re\left[ {K\left( f \right)} \right]$ is a function of the AC field frequency, $f$, via the equation:

$$\varepsilon_{x}^{*} = \varepsilon_{x} - \frac{{j\sigma_{x} }}{2\pi f}$$

where the subscript $x$ refers to the interior of the cell, the membrane or the medium, while $\sigma_{x}$ are the respective electric conductivities. $Re\left[ {K\left( f \right)} \right]$ can have either positive or negative values which means that the DEP force exerted on the particle can be directed in the same direction as the electric field gradient (situation called “positive DEP”), or in the opposite direction (“negative DEP”). The dependance of $Re\left[ {K\left( f \right)} \right]$ on the AC frequency is called a DEP spectrum. The AC field frequencies at which the DEP force is null^22^ are called “crossover frequencies” (CO frequencies).

Presently, DEP shows to be useful for: (i) characterization of various cell types (glioblastoma cells, neural stem cells); (ii) cell fusion; (iii) vaccine production; (iv) protein purification; (v) biological and medical applications which need cell separation (e.g., circulating tumor cells separation, cancer immunotherapy)^20,23–26^.

In this paper we propose a DEP-based method to determine the cell electric parameters (membrane and cytosol conductivity and permittivity) of human MSCs and osteogenic and adipogenic progenies. These electric parameters were computed based on DEP spectra measurements done at various moments of the differentiation process. Based on the computed parameters, a simulation of a microfluidic DEP cell separation process was conducted.

## Materials and methods

### Cell culture and differentiation

Human adipose-derived MSCs were isolated from surgical waste of female individuals (age between 23 and 37 years) undergoing elective lipoaspiration of waist and hips (after patient informed consent was obtained, in accordance with the French law Art.L. 1245-2 du Code de la Santé Publique). The cells were isolated according to procedures described by Guilak et al.^27^ and grown in DMEM high glucose with GlutaMAX (Gibco, 31966–047) supplemented with 10% fetal bovine serum (Sigma, F7524), 100 U/mL penicillin and 100 mg/mL streptomycin (Gibco, 15140122), at 37 °C in a humidified incubator with 5% CO_2_. Cells passage was done once or twice a week.

Differentiation was induced at passage 9, as previously described^18^. Briefly, prior to differentiation, cells were seeded at a density of 10,000 cells/cm^2^ and left in culture for 3–4 days to reach confluence. After reaching full confluence, the normal culture medium was removed and replaced with differentiation medium (this being considered the 1st day of differentiation). The osteogenic medium was composed of complete MEM α high glucose with GlutaMAX (Gibco, 32,561,037) supplemented with 100 nM of dexamethasone (Sigma, D2915), 200 μM of ascorbic acid (Sigma, 49752) and 10 mM of glycerol 2-phosphate (Sigma, 50020). The osteogenic differentiation lasted 4 weeks (during which the medium was changed twice a week). The adipogenic medium was composed of complete DMEM high glucose with GlutaMAX supplemented with 1 μM dexamethasone, 200 μM indomethacin (Sigma, I7378), 500 μM 3-isobutyl-1-methylxantine (Sigma, I5879) and 10 μg/mL insulin (Sigma, I9278). The adipogenic differentiation lasted 4 weeks (during which the medium was changed 3 times a week).

### Assessment of differentiation

Osteogenic cells were labelled with Alizarin Red staining according to the following protocol: cells were washed in PBS (Gibco, 10010023) and fixed in 95% methanol for 10 min. Alizarin Red S (Sigma, A5533) solution 2% in deionized water was added for 5 min, then the cells were rinsed multiple times with water until no eluting stain could be seen. Ca^2+^ deposits were observed under an epifluorescence microscope (Zeiss, Rueil-Malmaison, France). Osteogenic differentiation was also assessed using the alkaline phosphatase (ALP) assay (Supplementary information contains the method description and the results—Fig. S1B).

Adipogenic cells were stained with Bodipy: after the cell medium was removed, the cells were incubated for 30 min with 10 µM Bodipy (Sigma, 790,389) prepared in fresh medium. The cells were rinsed with fresh medium. The lipid droplets were visualized using the FITC filter of the epifluorescence microscope. Adipogenic differentiation was also assessed using the Oil Red O staining method (Supplementary information contains the method description and the results—Fig. S1A).

All assessments of cell differentiation were conducted in three biological replicates. To evaluate the differences among the groups, one-way ANOVA followed by Dunnett's multiple comparison test was employed.

### DEP spectra measurements

The cells were harvested using TrypLE (Gibco, 12604013). In the case of osteogenic cells, the first week, the cells were detached with TrypLE. For weeks 2 to 4, the upper layers were detached with Type I Collagenase (Gibco, 17018029) and the bottom layers were detached with TrypLE. In week 4 cells were pre-treated with EDTA (10 mM in PBS) to remove the calcium sediments.

The cell suspension (for all categories) was centrifuged at 300 × *g* for 5 min at 24 °C. The supernatant was discarded, and the pellet was washed twice with sucrose solution 300 mM (pH 7.4, ~ 0.01 S/m) without detaching it from the tube bottom. Finally, the pellet was resuspended in one of two different conductivity DEP buffers: 0.128 and 0.04 S/m. The 0.128 S/m buffer contains 250 mM Sucrose, 8 mM Na_2_HPO_4_, 2 mM KH_2_PO_4_, 1 mM MgCl_2_ (pH 7.4, 286 mOsm/kg). The 0.04 S/m buffer was obtained by mixing the 0.128 buffer with a solution of 300 mM sucrose (brought to pH 7.4, ~ 0.010 S/m) at a ratio of ~ 3:7. Cells suspended in different DEP buffers (at 5 × 10^6^ cells/mL) were further used for DEP spectra acquisition.

DEP spectra were acquired using a 3DEP dielectrophoretic analysis system (DEPtech, LABtech, UK) by using DEPwell 805 chips from the same producer. These chips have 20 wells with a system of incorporated electrodes, making it possible to simultaneously apply different frequencies in each well, in the range of 10 kHz–50 MHz. During the electric field application, sequencies of images of each well are acquired. From the analysis of cells distribution in each well, a dimensionless parameter called “relative DEP force”, directly proportional to the real part of the Clausius–Mossotti factor, $Re\left[K\left(f\right)\right]$, may be computed.

Spectra were acquired at 20 frequencies between 10 kHz and 40 MHz, with an acquisition duration of 60 s, the voltage applied to the electrodes being 10 V. Each experiment was repeated 48 times for the MSCs group (using cells originating from 6 flasks) and 24 times for each of the differentiated cells groups (using cells originating from 3 flasks for each group). Each spectrum was recorded using a fresh cellular suspension. Prior to spectra acquisition, the average dimension of cells was measured, using benchtop cell counter (Countess 3, Invitrogen, US).

### DEP spectra analysis

The DEP spectra were analyzed with the OpenDEP program which is an open-source program for simulating or analyzing DEP spectra (in-house developed, GitHub link: https://github.com/IoanTivig/OpenDEP_Compute.git, requirements to run in Python are specified in the root of the program, in the requirements.txt). OpenDEP allows the automation of data analysis and the automatic selection or elimination of spectra with an R^2^ below a defined value or outliers within the spectra. Output parameters of OpenDEP are membrane permittivity and conductivity, cytosol conductivity, and CO frequencies.

For the best fitting of acquired spectra, the input parameters used in OpenDEP are given in Supplementary information, Table 1.

The Tukey test was used for statistical analysis of the data using Python (scipy and numpy libraries).

### Simulations of DEP separation of cells

To simulate separations of MSCs from osteogenic or adipogenic cells in their different stages of differentiations, Autodesk Fusion 360 was used for the design of a microfluidic channel with DEP electrodes, and COMSOL Multiphysics for the actual simulation of electric fields, fluid flow and particle tracking (simulation in 2D, modules used: electric currents, fluid flow and particle tracing for fluid flow). The model of the microfluidic channel is presently theoretical, no physical prototype has been done yet. The input parameters are given in Supplementary information, Table 2.

Electrical parameters of cells were those computed based on the experimental DEP spectra. The buffer conductivity which gave the highest DEP force was chosen for the simulation (0.04 S/m). The frequency at which DEP separation was simulated was chosen equal to the first CO frequency of one cell line while the other cell line experienced a positive DEP. In the simulation, each particle was a unique one, having randomly attributed parameters ranging within the standard deviation of those computed using the DEP spectra fittings and cell size measurements (performed for each week of differentiation). Particles were introduced into the entrance of the microfluidic channel in a random pattern, to mimic as much as possible a real situation.

### Ethical approval

The human adipose-derived MSCs used in this study were the same cells batches as those used in the article published in Stem Cell Research and Therapy journal in 2018 (10 .1186 /s1328 7- 018 -0942-x) . These cells were surgical wastes and, as such, the French law Art.L. 1245-2 du Code de la Santé Publique established that there was no need to receive the authorization from an ethics committee.

## Results and discussion

### MSCs differentiation assessment

**Figure 1:**
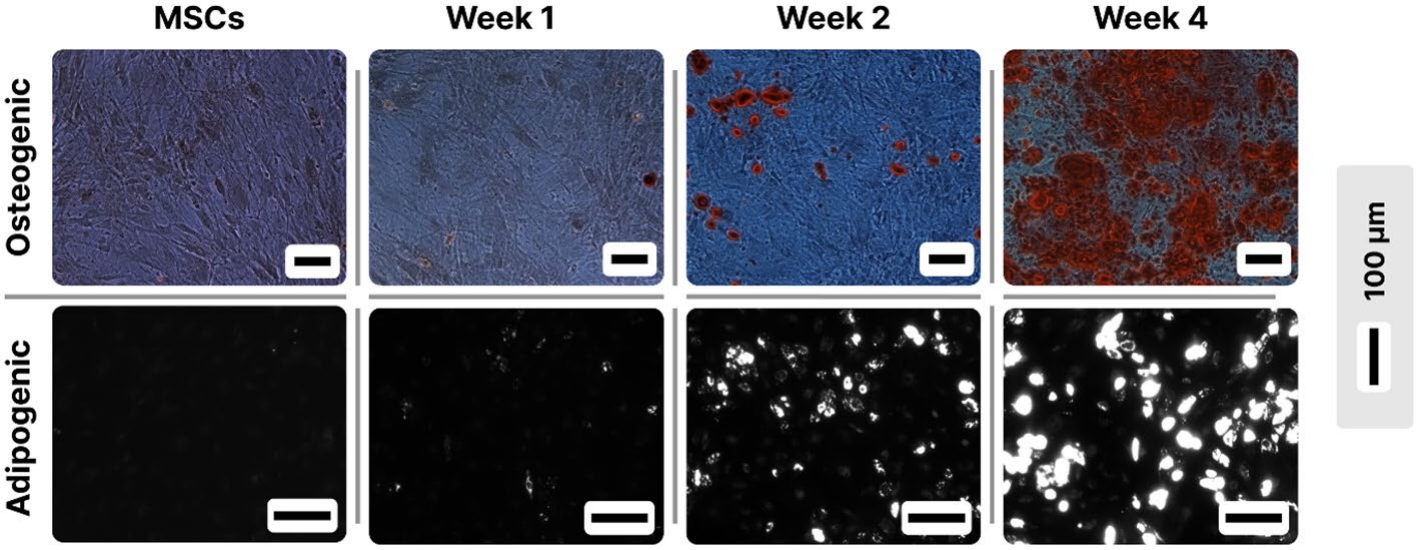
Microscopic evidence of MSCs transformation towards osteogenic (first column) and adipogenic cells (second column). For the osteogenic transformation cells were stained with Alizarin red and observed under phase contrast microscopy, where calcium deposits can be seen as black spots. For the adipogenic transformation, staining was done with Bodipy, and the formation of lipid droplets was observed under fluorescence microscopy as bright spots.

As shown in Fig. 1 in the first column (corresponding to osteogenic differentiation), calcium agglomerations were not observed after 1 week of differentiation but started to become visible in week two; in the fourth week the calcium agglomerations were covering more than a third of the optical field.

In the case of the adipogenic differentiation, the lipid droplets were hardly observed at week one in the differentiation medium (Fig. 1, second column). The process continued thereafter and in week four, droplets were present in about half of the cells.

The microscopic evaluation confirmed that, at the end of the fourth week, the cells were fully differentiated, in the sense that they attained the maximum differentiation state possible, considering the methods used here. The DEP experiments were thus performed weekly during their differentiation until the end of this time interval.

### DEP preliminary experiments

Considering that the phenotypic behavior of MSCs growing in their usual culture medium might be modified by cell confluence^28^, preliminary experiments were conducted to check whether these conditions affect the DEP spectrum.

For the MSCs used in this study (obtained from peritoneal adipose tissue), there were observable differences in the DEP spectra of high and low (~ 50%) confluence MSCs (Supplementary information Fig. S2A) which led to differences in the corresponding CO frequencies (Supplementary information Figs. S2B and C). Further experiments were done using high-confluence MSCs cultures since their differentiation protocols imply such conditions.

To optimize the experimental conditions concerning the DEP buffer conductivity, DEP spectra were acquired using the conductivities: 0.04 S/m (Supplementary information Fig. S3 black traces) and 0.128 S/m (Supplementary information Fig. S3 grey traces). As expected, at higher conductivity, the first CO frequency was significantly shifted towards higher values, no matter the cell type. Moreover, in the case of differentiated cells, this shift was so important that a robust fit could not be done anymore (within the used frequencies domain, the real part of Clausius–Mossotti factor either did not reach the plateau or hardly tended to go down to the second CO frequency). Based on this observation, further experiments were done in 0.04 S/m buffer conductivity.

**Figure 2:**
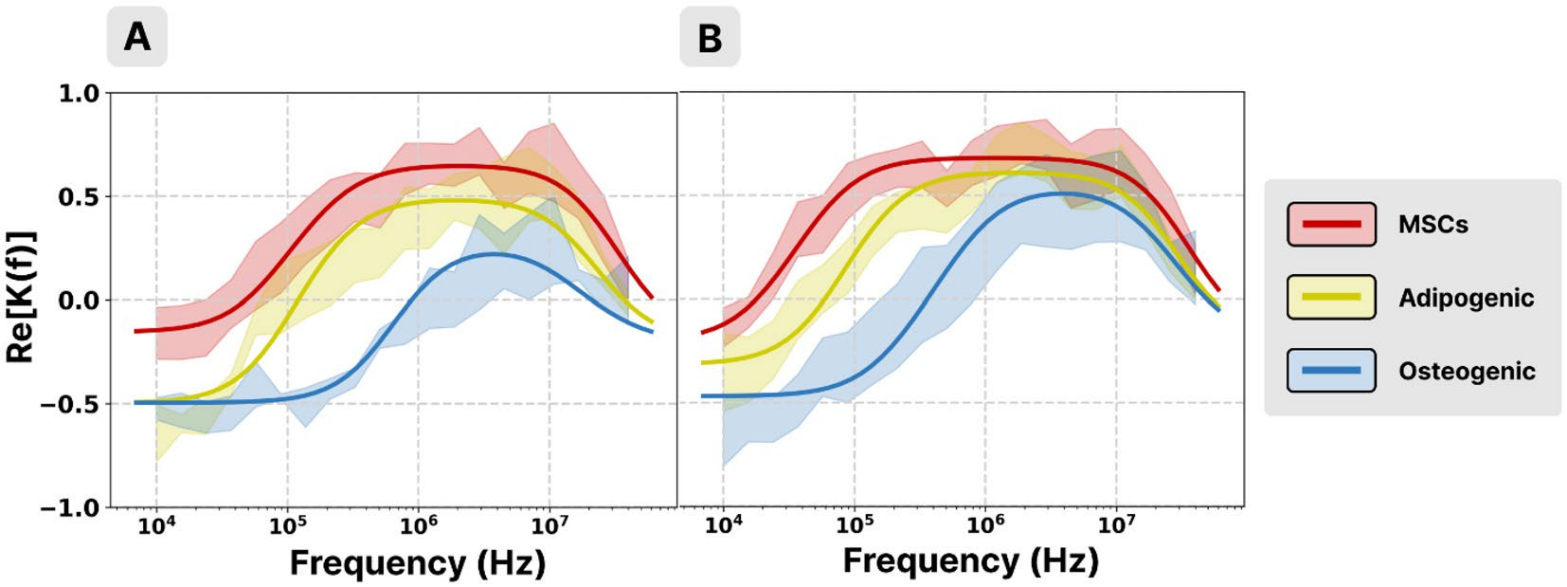
DEP spectra of MSCs (red trace), fully differentiated adipogenic (yellow trace) or osteogenic (blue trace) cells obtained from two donors (**A** and **B**). Standard deviations are represented as low opacity areas.

When comparing DEP spectra from MSCs and fully differentiated osteogenic or adipogenic cells coming from two donors, it was observed that there were differences between DEP spectra of MSCs obtained from these donors (Fig. 2). Despite this variability, modifications of DEP spectra due to the differentiation into either osteogenic or adipogenic, were similar. Once the cells were differentiated, the first CO frequency was shifted towards higher values, the plateau value diminished, and the second CO frequency lowered.

These systematic modifications of DEP spectra ensured the reliability of further DEP analysis and of the cell electric parameters characterization consecutive to the differentiation process.

### DEP analysis of differentiated MSCs

**Figure 3:**
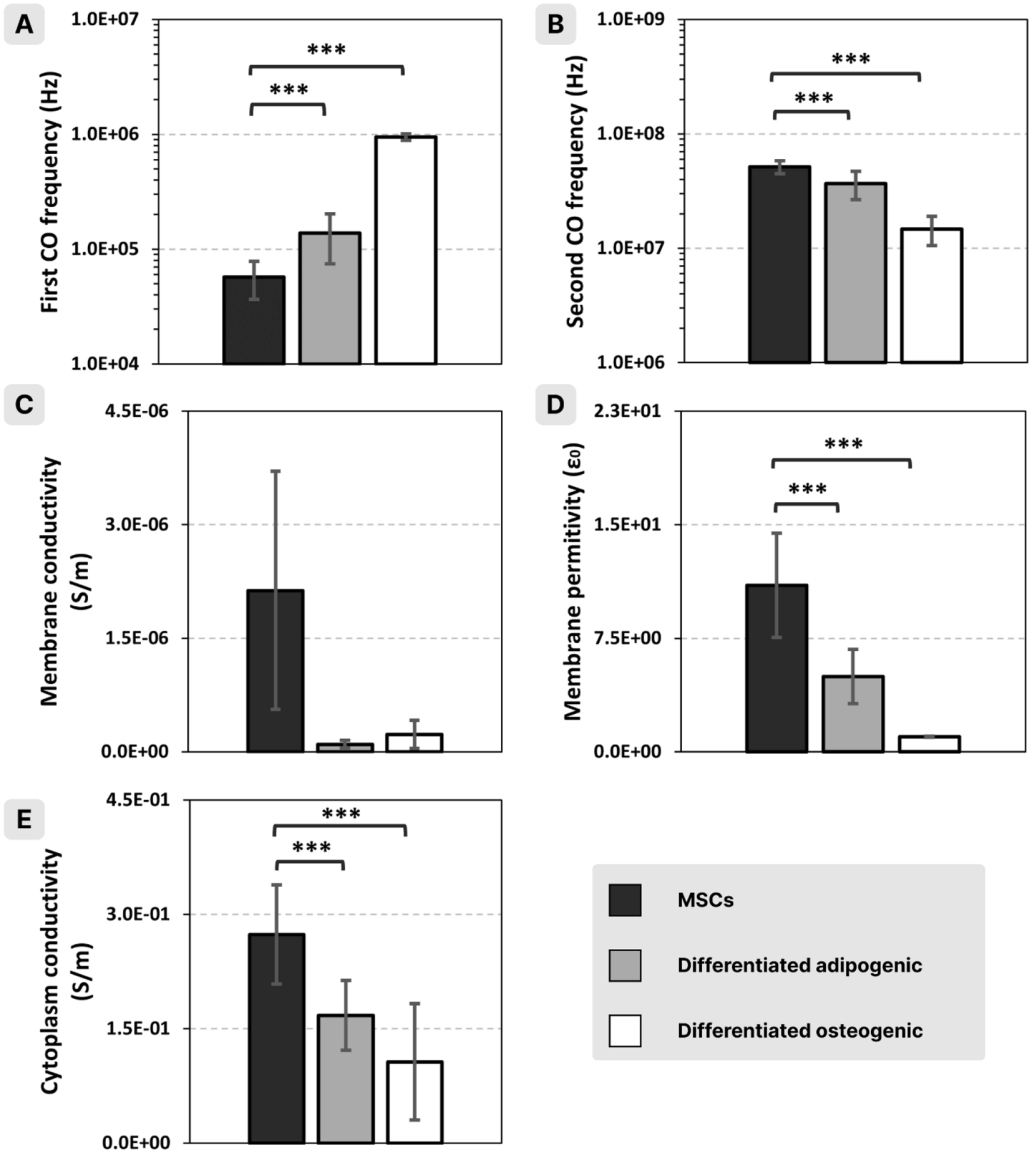
Computed CO frequencies (**A** and **B**), and electric parameters (**C**, **D**, **E**) of MSCs (black), fully differentiated adipogenic (grey), and fully differentiated osteogenic (white) cells from the same donor (*, **, *** for *p* < 0.05, 0.01, 0.001, respectively).

The analysis of the behavior of MSCs and fully differentiated cells was performed on spectra acquired from cells coming from the same single donor. Then the same analysis was performed for the cells from a second donor (Supplementary information Fig. S4). Important differences were found in the shape of the spectra, that are reflected in the CO frequencies and the plateaus values (both negative and positive) (Fig. 2A). For both differentiated cells, the first CO frequency shifted towards higher values compared to nondifferentiated cells (Fig. 3A), while the second CO frequency shifted towards lower values (Fig. 3B).

Electric parameters computed based on these spectra also showed significant differences: there was a decrease in membrane conductivity, membrane permittivity, and cytoplasm conductivity for both types of differentiated cells as compared to MSCs (Fig. 3C,D, and E). In the case of membrane permittivity and cytoplasm conductivity, the decrease was more pronounced for the osteogenic cells.

Similar values were found on *Neurospora crassa* slime cells using DEP^29^ (membrane relative permittivity 8.3–9.4 and membrane conductivity < 1–2.2 × 10^–6^) or dielectric spectroscopy^30^ (membrane relative permittivity 7.9 and cytosol conductivity 0.23 S/m). When working on Myeloma Tib9 cells, Marszalek and col.^29^ found a membrane relative permittivity of 9.4 and a membrane conductivity of less than 2 × 10^–6^. More recently, using optically induced dielectrophoresis, Liang and col.^31^ also found similar values working on Raji, MCF-7, and HEK293 cells: membrane relative permittivities were in the range 6–9 and membrane conductivities between 0.7 and 5 × 10^–6^ S/m.

Regarding studies on human MSCs, using dielectrophoresis, Adams and col.^32^ found a relative membrane permittivity in the range of 0.79 and 1.1, a membrane conductivity of 1 × 10^–6^ S/m and a cytosol conductivity of 0.5 S/m. All these values are in the range of those obtained in the experiments described in this paper.

### Evolution of cellular electric properties during the cell differentiation process

**Figure 4:**
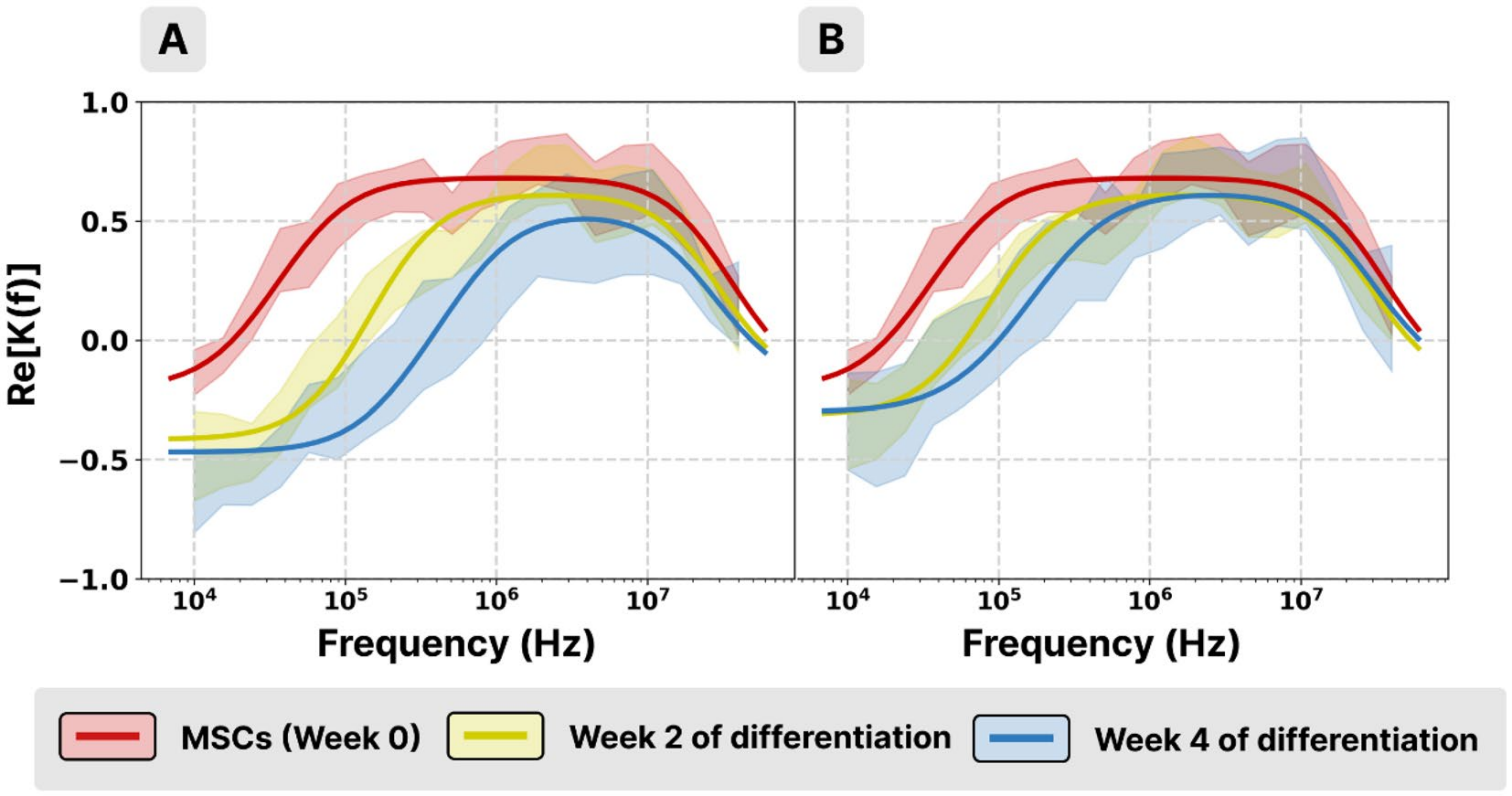
DEP spectra of osteogenic (**A**) and adipogenic (**B**) cells from the same donor as a function of the number of weeks in the differentiation process: MSCs (red), week 2 (yellow) and week 4 (blue). Standard deviations are represented as low opacity areas.

The DEP spectra acquired at different moments of the differentiation process showed an important increase of the first CO frequency both in the case of the osteogenic and adipogenic transformations (Fig. 4A and B).

**Figure 5:**
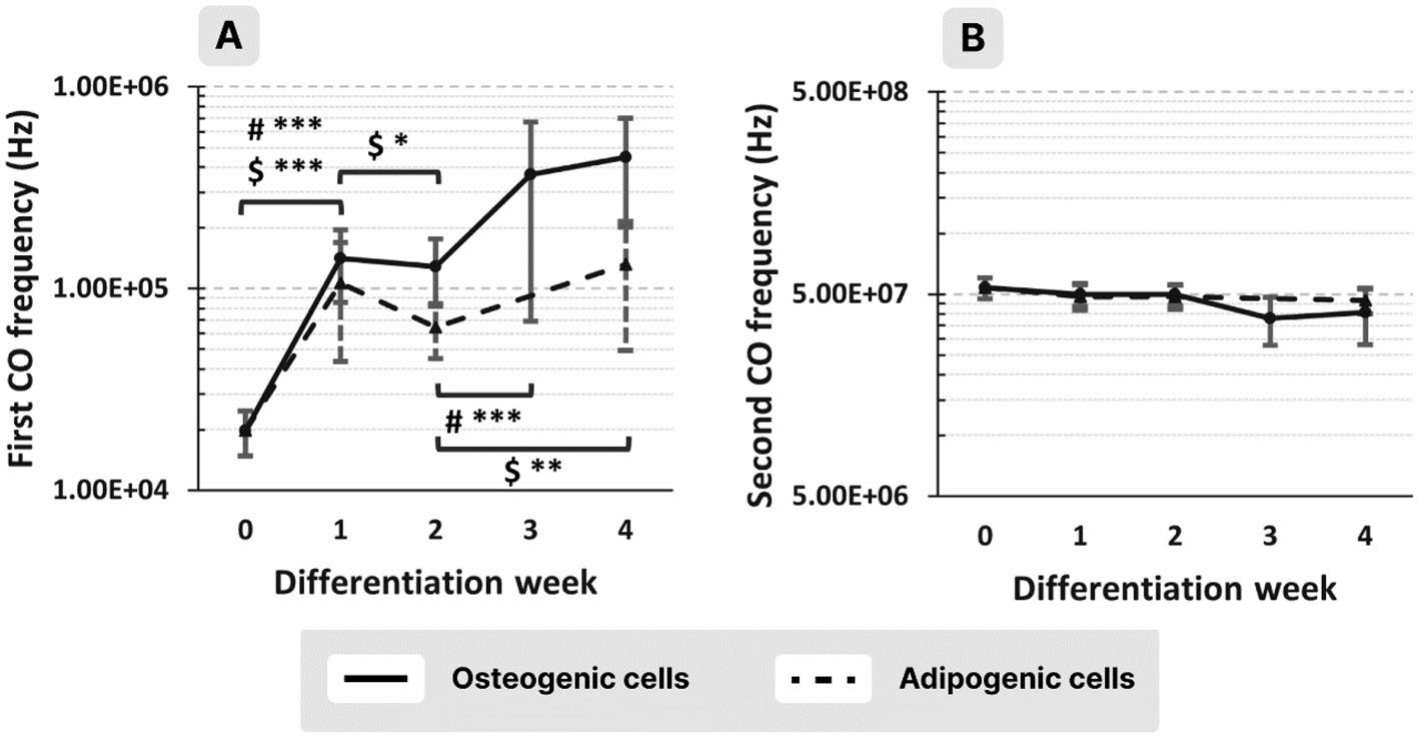
First (**A**) and second (**B**) CO frequencies of osteogenic (continuous trace) and adipogenic (dashed trace) cells from the same donor as a function of the week of differentiation (*, **, *** for *p* < 0.05, 0.01, 0.001, respectively; #, $ refers to osteogenic and adipogenic cells, respectively).

In the case of the osteogenic cells, the first CO frequency showed an important increase after the first week and then it continued to slowly increase as the cells progressed in the differentiation process. The adipogenic cells had the same behavior during the first week of differentiation and then, the first CO frequency remained almost constant (Fig. 5A).

However, the second CO frequency presented a very low tendency (no statistically significant difference) of decreasing, especially in the last 2 weeks of the differentiation period and for both types of differentiation (Fig. 5B).

The observed shifts of the spectra are a consequence of the modifications of various electric parameters of the cells during their differentiation.

**Figure 6:**
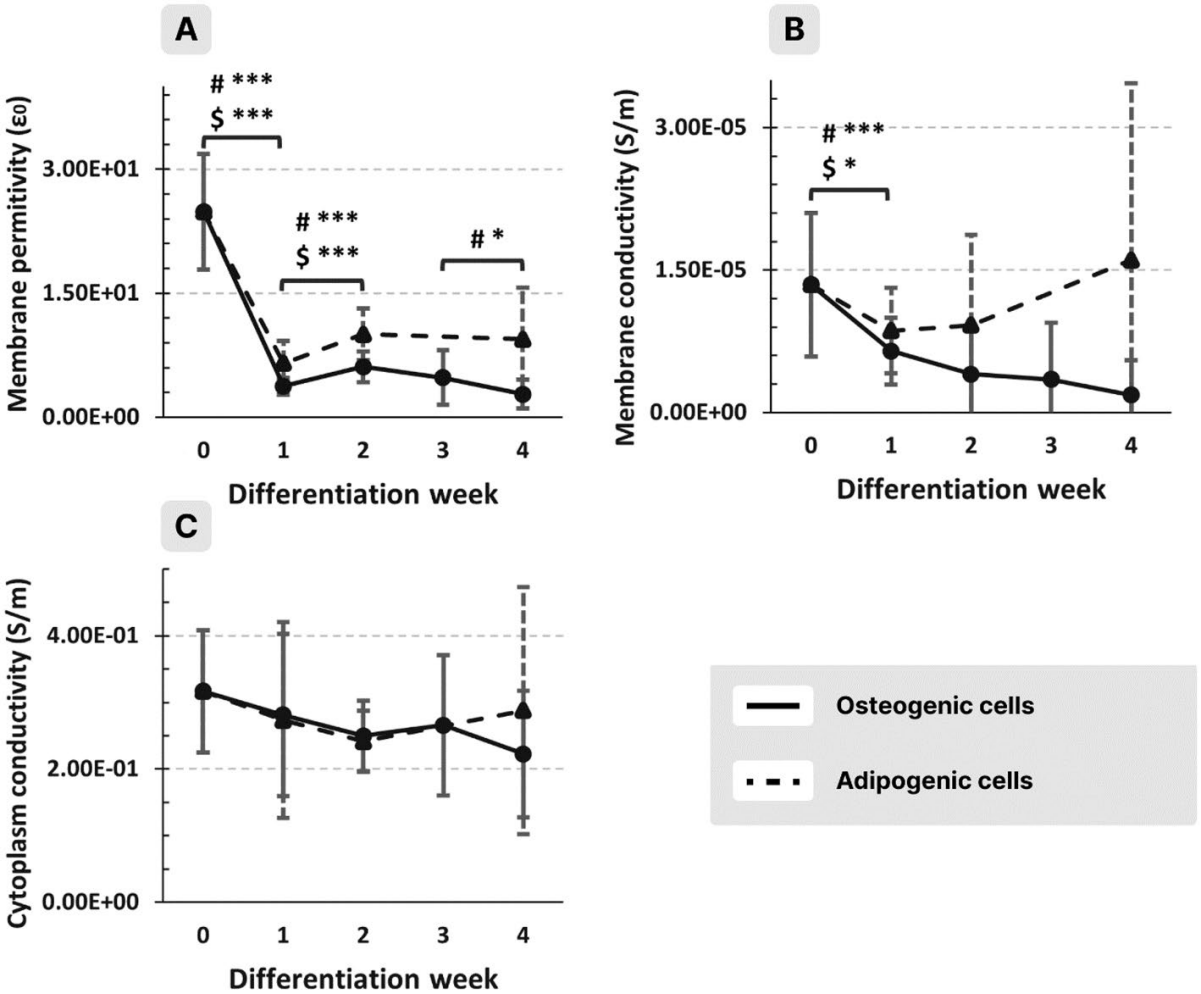
Membrane permittivity (**A**), membrane conductivity (**B**), and cytoplasm conductivity (**C**) of osteogenic (continuous trace) and adipogenic (dashed trace) cells from the same donor as a function of the week of differentiation (*, **, *** for *p* < 0.05, 0.01, 0.001, respectively; #, $ refers to osteogenic and adipogenic cells, respectively).

The membrane permittivity of the cells dropped in the first week and then remained constant for both cell types (Fig. 6A). The membrane conductivity of osteogenic cells decreased slowly during the whole period of transformation (Fig. 6B continuous trace), while in the case of the adipogenic cells (Fig. 6B dashed trace), it decreased only in the first week and then showed a slight increasing tendency. The cytoplasm conductivity of both cell types slightly decreased during the process (Fig. 6C).

It is interesting that the cell membrane permittivity decreases abruptly in the first week of transformation even before cells exhibit observable morphologic changes (Fig. 1). It has been demonstrated^33^ that membrane permittivity is influenced by its phospholipid composition rather than the presence/absence of proteins. The hydrophobic core of the membrane consists of hydrocarbon tails of the phospholipids and hydrophobic segments of the integral membrane proteins. As concerns the phospholipids, the membrane of differentiated MSCs shows different compositions^34^: when compared to the non-differentiated MSCs, higher concentrations of phosphatidylserine and N-acylphosphatidylethanolamine were mainly observed for osteogenic differentiated MSCs, and of phosphatidylcholine for adipogenic differentiated MSCs. This change in lipid composition may be associated with the drop in permittivity observed in Fig. 6 A. As concerning membrane proteins or cholesterol, their influence on membrane permittivity^33,35^ is too small to contribute significantly to the observed drop in the first week.

### Simulations of DEP separation of cells

**Figure 7:**
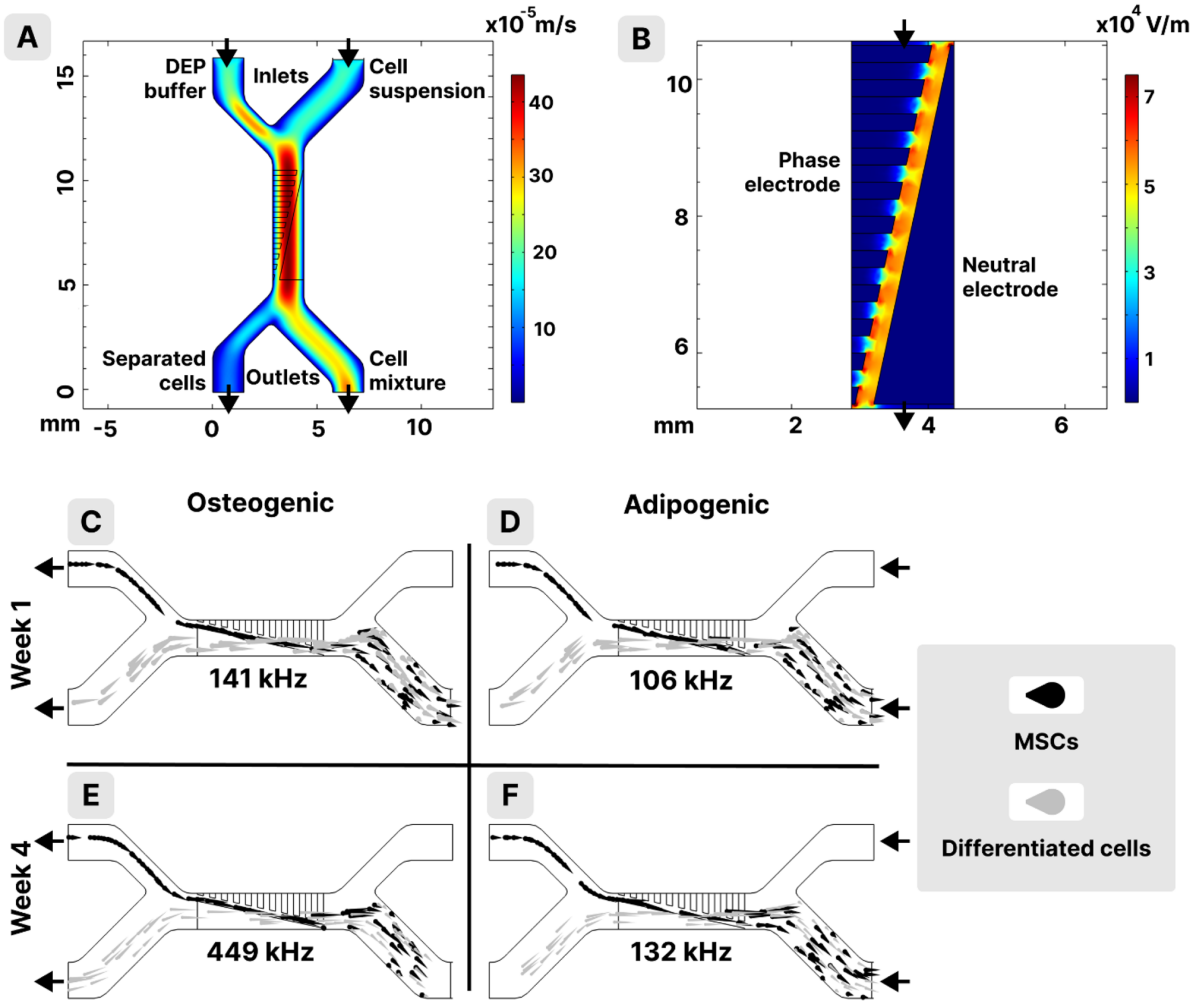
Simulations of the DEP based microfluidic separation device. (**A**) Velocity of the cell suspension and DEP buffer, (**B**) electric field intensity distribution along the central channel. In (**C**–**F**) black comets represent undifferentiated MSCs, and grey comets represent differentiated MSCs. (**C**) MSCs mixed with osteogenic cells at the first week of differentiation, (**E**) MSCs mixed with fully differentiated osteogenic cells, (**D**) MSCs mixed with adipogenic cells at the first week of differentiation, (**F**) MSCs mixed with fully differentiated adipogenic cells. Size distribution and electrical parameters of cells used in the simulations were within measured ranges (Supplementary information Table 2).

The variations of the electric parameters associated with early-stage cell differentiation can be exploited for a DEP-based cell separation aiming to collect those cells which started their transformation. A model of such a microfluidic device is further presented. The geometry of the separation device and the simulated velocity inside the microfluidic channels can be seen (Fig. 7A). There are two inlets (on top of the model) and two outlets (on the bottom of the model). The cellular suspension is pumped inside the microfluidic device through the right-side inlet. The purpose of the left-side inlet (through which DEP buffer is pumped) is to push all cells towards the right side of the central channel, from where cells are exposed to the DEP field all along the separation channel (Fig. 7B).

The DEP field is given by two electrodes, both located on the bottom of the central channel: a continuous electrode with an oblique profile (neutral electrode, on the right side in Fig. 7B) and a finger-like profile electrode (phase electrode, on the left side in Fig. 7B). This geometry gives an electric field inhomogeneity which follows a path positioned diagonally to the axis of the channel (where the field intensity is the highest), from the right wall to the left wall. Thus, cells which feel the DEP force (either positive or negative) will travel towards the left side of the microfluidic channel (cells subject to pDEP will gather on the path and cells subject to nDEP will avoid crossing the path and will stay thus on the left side of the channel). At the bottom split of the central channel, cells that were dielectrophoretically pushed to the left side will exit through the left side outlet, and cells which did not feel any DEP force (cells that are at CO frequency) will travel undisturbed and exit through the right-side outlet. We used a 2D simulation with ultra-thin electrodes. In this setup, cells of interest flowing close to the channel's bottom are experiencing the electric field and will be separated, while others (at cross-over or on a higher height) will exit on the right outlet. The main limitation of the 2D simulation is that it might overestimate the separation yield rather than the purity of the target cell suspension.

In Fig. 7C–F simulations for the separation of MSCs and differentiated osteogenic (C and E) or adipogenic (D and F) cells can be seen. The simulation shows a successful separation for both cell types (with 100% purity), no matter if they were fully differentiated (Fig. 7E and F) or just in their first week of differentiation (Fig. 7C and D). Interestingly, cells engaged in the differentiation process with similar sizes to MSCs (as they are in their first week of differentiation, either adipogenic or osteogenic) can be efficiently separated only due to differences in their membrane permittivity (which is reflected specifically in the value of the first CO of DEP spectra^36^).

The possibility of a DEP separation opens the path towards two possible applications: purification of differentiated cells from a heterogenous population (a population which still has cells partially differentiated) and separation of MSCs from other cells originating from a source tissue. The advantages of using such a separation method rely on the fact that it can be done from the very beginning of the cell differentiation (i.e., in the first week of treatment) without the need of any cell labeling; moreover, the method requires low cost equipment and consumables (standard equipment and chemicals for cell biology laboratory and 3D printing are needed).

## Conclusions

Electrical parameters of MSCs and their osteogenic and adipogenic progenies have been assessed by DEP at various stages of the cell differentiation process. Most important modification of these parameters (a significant decrease of the membrane permittivity and conductivity) occurred after the first week of differentiation for both cell progenies. These modifications preceded the observable morphological changes due to differentiation and are associated with the biochemical characteristics of the transformed cells (Supplementary information Fig. S1). During the following weeks, until cells became fully differentiated, only the membrane permittivity continued to slowly decrease. The modification of the electrical parameters of the cells at the end of the first week of differentiation can thus be interpreted as an indicator of the beginning of the transformation process.

When employing the measured electrical parameters of MSCs and their progenies, we successfully modeled the separation process using a suitable DEP microfluidic device model. The simulations showed that the separation of cells of interest is achievable irrespective of the cell type or the degree of differentiation (first or last week).

Two possible applications are evident: the separation of differentiated MSCs in their early stage of differentiation, and the purification of MSCs suspensions of various “contaminant” cells collected from a source tissue.

### Supplementary Information

Supplementary Information.

## Data Availability

Experimental raw data are available upon request to the corresponding author.
