# Supplementary material for: Early differentiation of mesenchymal stem cells is reflected in their dielectrophoretic behavior
Source: Sci Rep. 2024 Feb 21;14:4330. doi: 10.1038/s41598-024-54350-z (PMC10881469; doi:10.1038/s41598-024-54350-z)
Supplement: Supplementary file 1 — Supplementary Information. [file 41598_2024_54350_MOESM1_ESM.docx]

**Supplementary information for**

**Early differentiation of mesenchymal stem cells is reflected in their dielectrophoretic behavior**

Ioan Tivig, Leslie Vallet, Mihaela G. Moisescu, Romain Fernandes, Franck M. Andre,

Lluis M. Mir, Tudor Savopol

| Parameters | Values and units | Source of information |
| --- | --- | --- |
| Frequency | 10 kHz to 40 MHz | Recommended by 3DEP manufacturer |
| Electric potential | 10 Volts | Recommended by 3DEP manufacturer |
| Buffer permittivity | 78 | Recommended by 3DEP manufacturer |
| Buffer conductivity | 0.04 – 0.128 S/m | Chosen based on preliminary tests |
| Cell radius | MSCs: 9.1 – 11.4 μm  Osteogenic:  Week 1: 7.4 – 7.6 μm  Week 2: 6.6 – 7.4 μm  Week 4: 6.1 – 6.8 μm  Adipogenic:  Week 1: 9.6 – 10.1 μm  Week 2: 8.9 – 9.5 μm  Week 4: 6.3 – 8.6 μm | Measured |
| Cell membrane thickness | 6 nm | Reported in literature (1, 2) |

**Table 1.** Parameters used for analysis of DEP spectra and computation of cells electrical parameters.

| Parameters | Values and units | Source of information |
| --- | --- | --- |
| Channel height | 500 μm | Chosen based on model |
| DEP field frequency | 100 kHz – 450 kHz | The range covers CO frequencies as obtained from measured DEP spectra |
| Electric potential | 15 Volts | Chosen based on model |
| Fluid velocity at inlets | 150 μm/s | Chosen based on model |
| Fluid pressure at outlets | 0 Pa | Chosen based on model |
| Buffer permittivity | 78 | Recommended by 3DEP manufacturer |
| Buffer density | 1000 kg/m³ | Recommended by COMSOL developers |
| Buffer dynamic viscosity | 0.001 Pa·s | Recommended by COMSOL developers |
| Buffer conductivity | 0.04 S/m | Chosen based on preliminary test |
| Cell density | 1050 kg/m³ | Recommended by COMSOL developers |
| Cell radius | MSCs: 9.1 – 11.4 μm  Osteogenic:  Week 1: 7.4 – 7.6 μm  Week 4: 6.1 – 6.8 μm  Adipogenic:  Week 1: 9.6 – 10.1 μm  Week 4: 6.3 – 8.6 μm | Measured for each cell type and week of differentiation |
| Cell membrane thickness | 6 nm | Reported in literature (1, 2) |
| Cell membrane permittivity | 2.79 – 24.85 | Obtained from measured DEP spectra depending on the cell type and week of differentiation |
| Cell membrane conductivity | 1.84 x 10^-6^ – 1.60 x 10^-5^ | Obtained from measured DEP spectra depending on the cell type and week of differentiation |
| Cell cytoplasm conductivity | 0.22 – 0.32 S/m | Obtained from measured DEP spectra depending on the cell type and week of differentiation |

**Table 2.** Parameters used for simulations of cells separation.


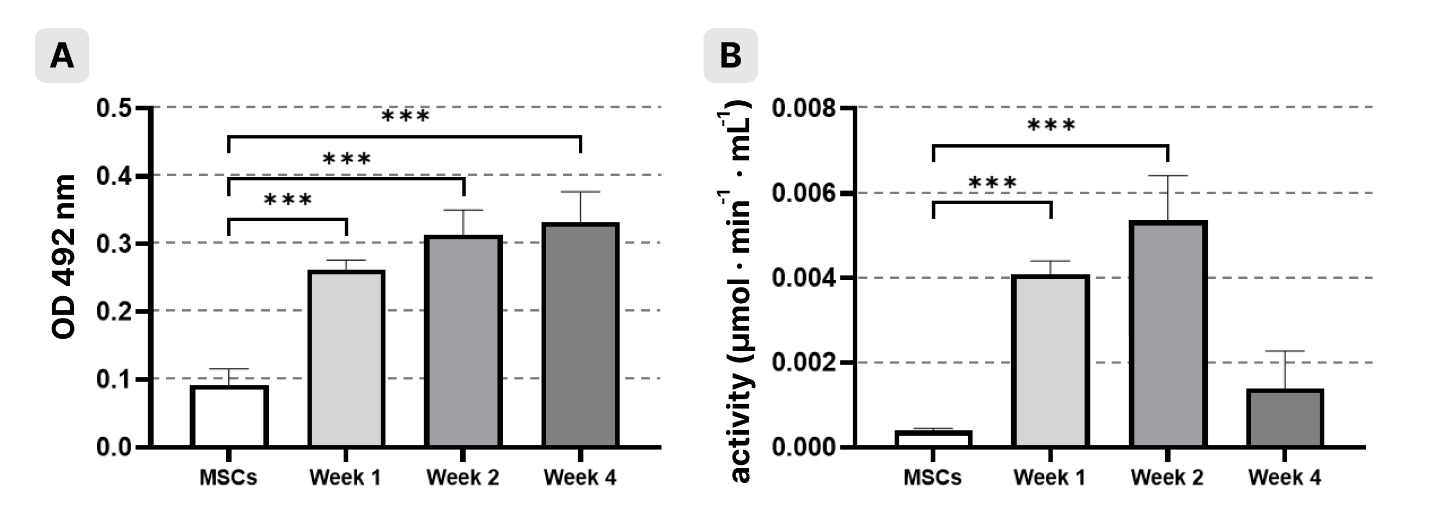


**Fig. S1.** Biochemical tests for MSCs differentiation at different moments after induction. (A) Oil Red O test for adipogenic differentiation, (B) alkaline phosphatase assay for osteogenic differentiation.

**Oil Red O staining (adipogenic differentiation)**

Prior to staining, cells were washed in PBS (Gibco, 10010023) and fixed in 10% neutral buffered formalin (Sigma, F8775) for 20 min at room temperature. The staining solution was prepared combining three volumes of Oil Red O (Sigma, O0625) stock solution at 5 g/L in isopropanol with two volumes of deionized water. Fixed cells were washed twice with deionized water and incubated with isopropanol/H2O (60/40) for 5 min at room temperature. Cells were then incubated with the staining solution for 20 min at room temperature. Cells were subsequently washed with deionized water until no eluting staining could be observed. Stained lipid vesicles were observed under an epifluorescence microscope (Zeiss, Rueil-Malmaison, France). The Oil Red was then extracted by incubating the cells with isopropanol and the absorbance of the eluted stain was spectrophotometrically measured at 492 nm (Tecan, Lyon, France).

**Alkaline phosphatase assay (osteogenic differentiation)**

Cells were harvested using TrypLE (Gibco, 12604013) (week 1 of differentiation), trypsin and collagenase I (Gibco, 17018029) (4 mg/mL) (week 2 to 4). A pre-treatment with EDTA (10 mM in PBS) was necessary to remove calcium deposits for week 4. The harvested cells were centrifuged at 300 × g for 5 min, room temperature, the pellet was resuspended in the assay buffer (Abcam, ab83369) at a concentration of 5 × 10^5^ cells/mL. Then, a centrifugation at 13,000 × g was done for 15 min at 4 °C. The supernatant was transferred to a new tube. 80 μL of supernatant were distributed in a 96-well plate. The background was measured in the wells where 50 μL of p-nitrophenyl phosphate (pNPP) 5 mM solution (Abcam, ab83369) and 20 μL of stop solution (Abcam, ab83369) were added simultaneously on top of supernatant. In the rest of wells, 50 μL of pNPP 5 mM was added on top of supernatant and incubated for 60 min at 25°C. Then 20 μL of stop solution was added. The optical density at 405 nm was measured (Tecan, Lyon, France). The quantity of p-nitrophenol (pNP) in each well was determined using a standard curve established using pNPP in known quantities and purified alkaline phosphatase enzyme (Abcam, ab83369).


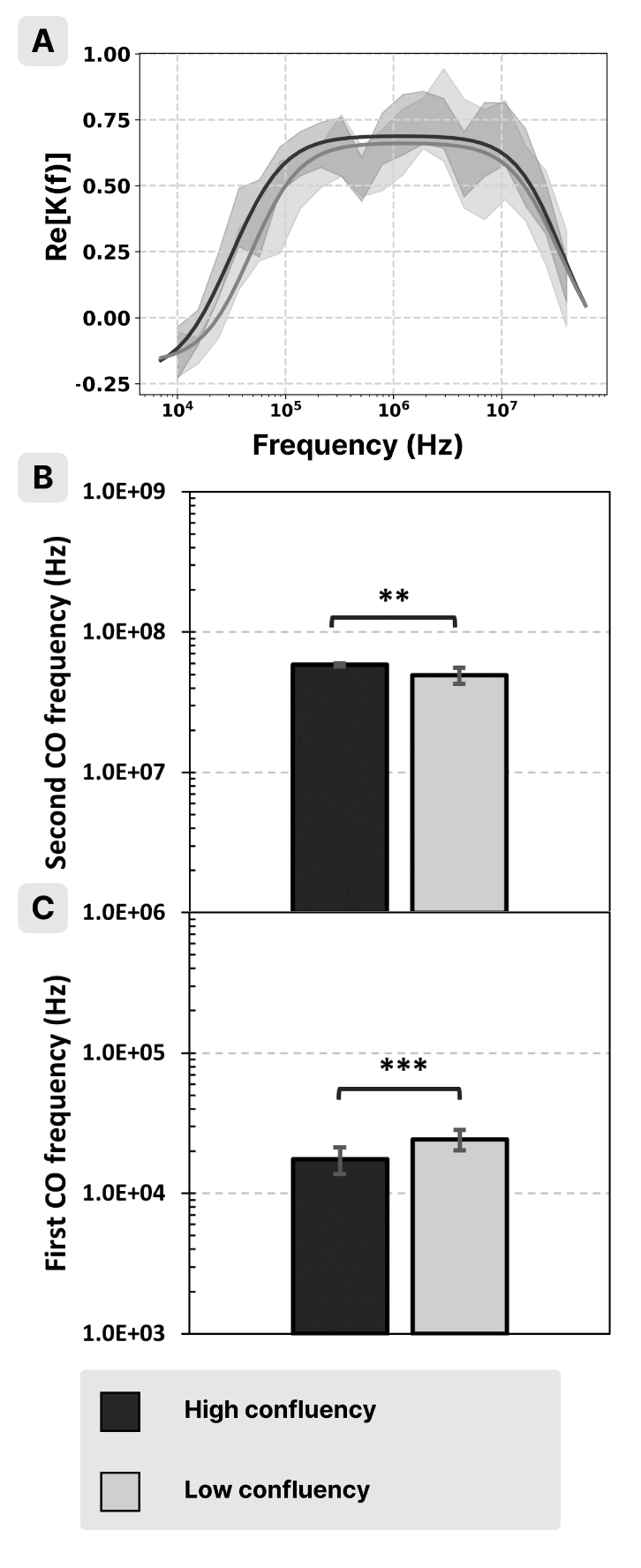


**Fig. S2.** DEP spectra of MSCs at high confluency (black) and low confluency (grey) (A), standard deviations are represented as low opacity areas; first and second CO frequencies (B and C) for MSCs at high confluency (black bar) and low confluency (grey bar) (**, *** for p < 0.01, 0.001, respectively). The cells belong to a single experimental batch.


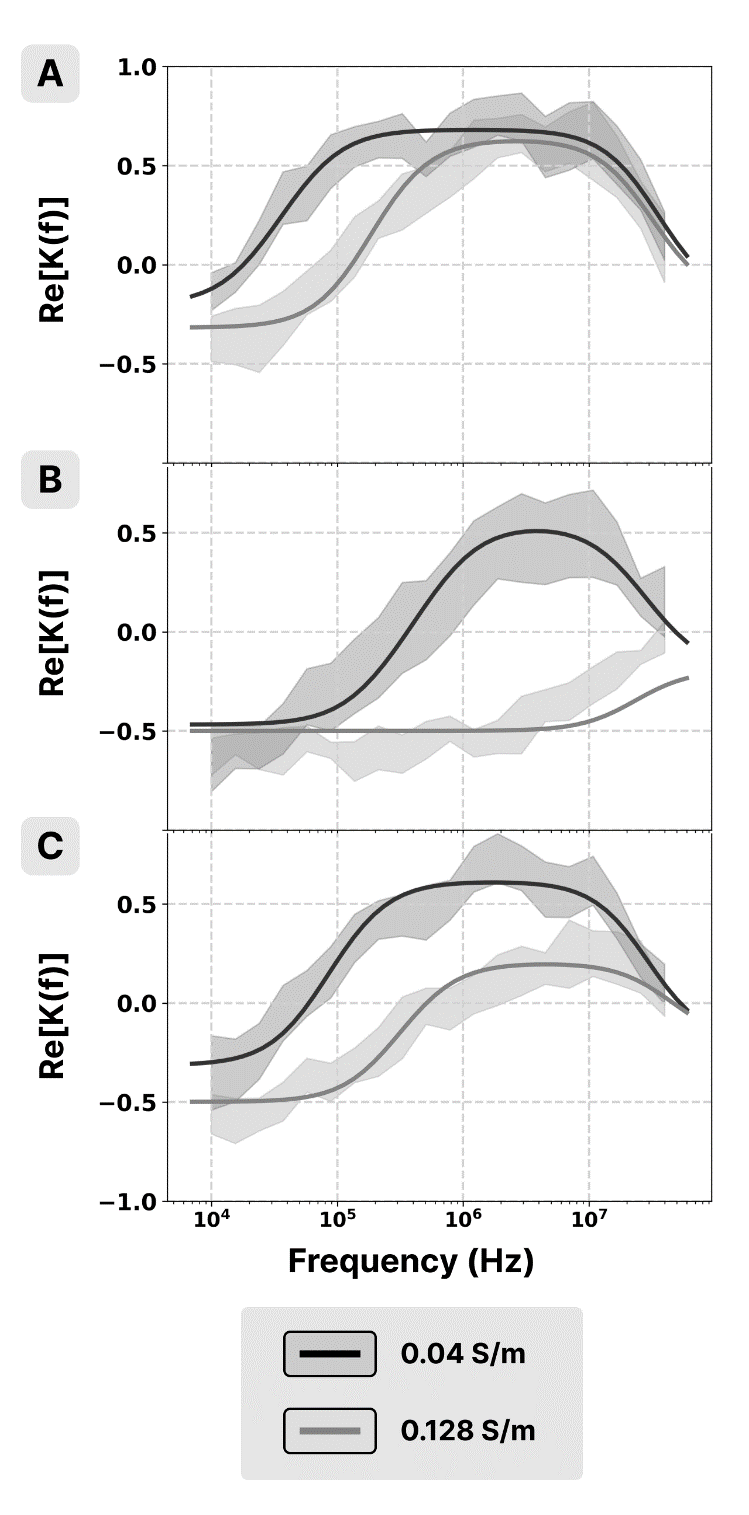


**Fig. S3.** DEP spectra of MSCs (A), differentiated osteogenic cells (B), and differentiated adipogenic cells (C), in DEP buffers of 0.04 and 0.128 S/m (black and grey traces, respectively). Standard deviations are represented as low opacity areas.


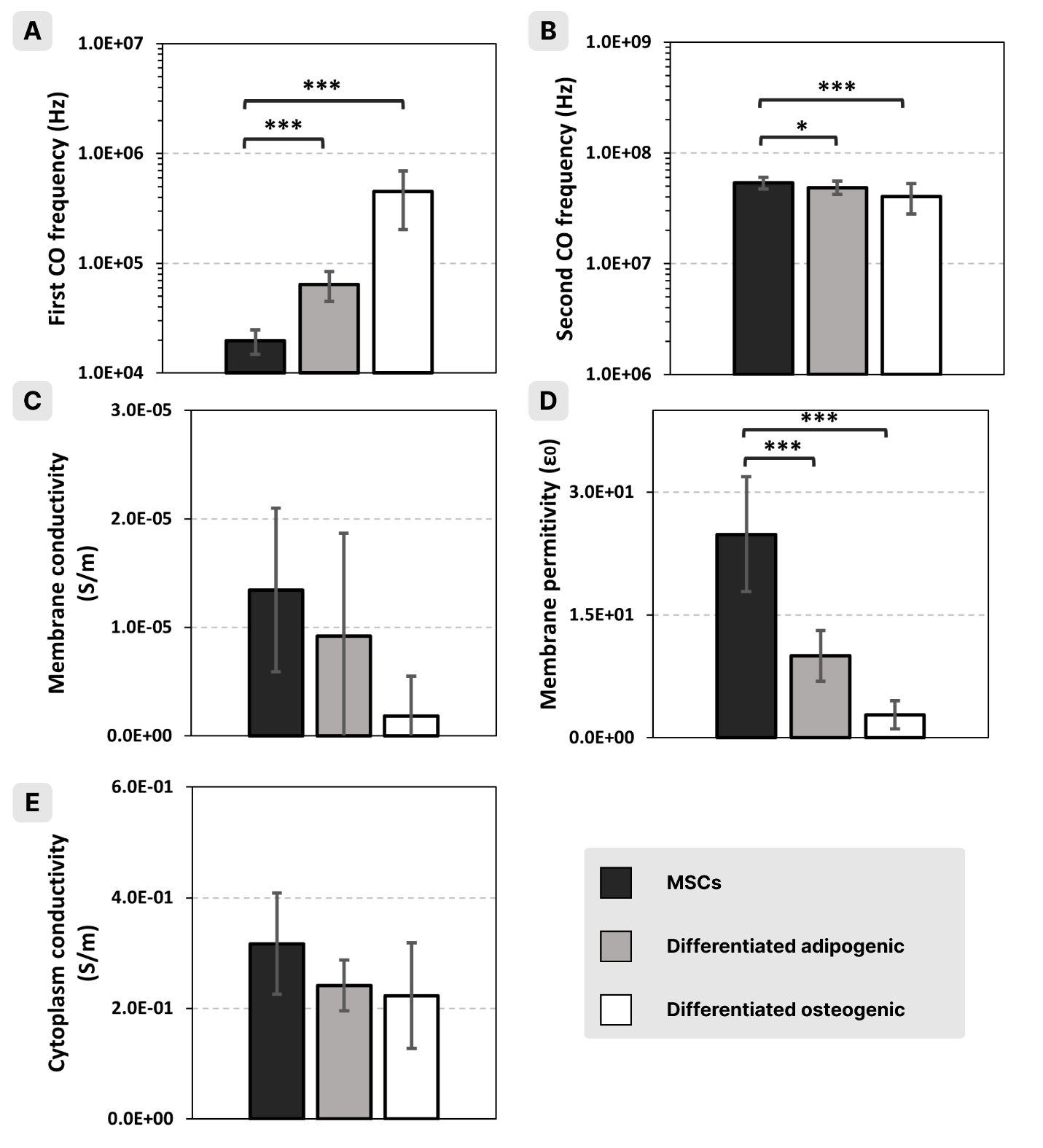


**Fig. S4** Computed CO frequencies (A and B), and electric parameters (C, D, E); MSCs (black), 4 weeks differentiated adipogenic (grey), and osteogenic (white) cells from the second donor (*, **, *** for p < 0.05, 0.01, 0.001, respectively).

**Bibliography**

1. Chen, T., Ghosh, A., & Enderlein, J. (2022). Cholesterol-Induced Nanoscale Variations in the Thickness of Phospholipid Membranes. *Nano Letters*, *23*, 2421–2426. https://doi.org/10.1021/acs.nanolett.2c04635
2. Cottet, J., Fabregue, O., Berger, C., Buret, F., Renaud, P., & Frénéa-Robin, M. (2019). MyDEP: A New Computational Tool for Dielectric Modeling of Particles and Cells. *Biophysical Journal*, *116*(1), 12–18. https://doi.org/10.1016/j.bpj.2018.11.021
